# Validating the use of a smartphone app for remote administration of a fear conditioning paradigm

**DOI:** 10.1016/j.brat.2019.103475

**Published:** 2019-12

**Authors:** K.L. Purves, E. Constantinou, T. McGregor, K.J. Lester, T.J. Barry, M. Treanor, M. Sun, J. Margraf, M.G. Craske, G. Breen, T.C. Eley

**Affiliations:** a King's College London, Social, Genetic and Developmental Psychiatry Centre, Institute of Psychiatry, Psychology & Neuroscience, London, UK; b School of Psychology, University of Sussex, Brighton, Sussex, UK; c Experimental Psychopathology Lab, Department of Psychology, The University of Hong Kong, Hong Kong; d Department of Psychology, University of California, Los Angeles, CA, USA; e Mental Health Research and Treatment Center, Rurh-University Bochum, Bochum, Germany; f NIHR Biomedical Research Centre for Mental Health, South London and Maudsley NHS Trust, London, UK

**Keywords:** Conditioned fear, Anxiety, Smartphones, Methodology, Computerized assessment, Psychometrics

## Abstract

Fear conditioning models key processes related to the development, maintenance and treatment of anxiety disorders and is associated with group differences in anxiety. However, laboratory administration of tasks is time and cost intensive, precluding assessment in large samplesnecessary for the analysis of individual differences. This study introduces a newly developed smartphone app that delivers a fear conditioning paradigm remotely using a loud human scream as an aversive stimulus. Three groups of participants (total n = 152) took part in three studies involving a differential fear conditioning experiment to assess the reliability and validity of a smartphone administered fear conditioning paradigm. This comprised of fear acquisition, generalisation, extinction, and renewal phases during which online US-expectancy ratings were collected during every trial with evaluative ratings of negative affect at three time points. We show that smartphone app delivery of a fear conditioning paradigm results in a pattern of fear learning comparable to traditional laboratory delivery and is able to detect individual differences in performance that show comparable associations with anxiety to the prior group differences literature.

## Highlights

• A smartphone app reliably delivers a differential fear conditioning paradigm using an aversive human scream.
• Within-person associations of fear conditioning between laboratory and app delivery do not differ from those across time.
• App-based administration is able to detect individual differences in anxiety related processes.
• Fear conditioning data can be collected with minimal financial and time cost.

## 1 Introduction

Fear conditioning paradigms model associative learning processes that are implicated in the development and maintenance of anxiety disorders and extinction-based treatments. Indeed, the Pavlovian extinction of fear served as the basis for exposure-based treatment of anxiety disorders (Bouton, 1988). Differential fear conditioning refers to a paradigm presenting two stimuli, one of which is reinforced by an aversive outcome (unconditional stimulus; US). Consistent differences between individuals with anxiety disorders and healthy controls during differential fear conditioning paradigms provide diagnostic and construct validity in that it demonstrates that the model may be a useful diagnostic marker, and is disease relevant (Vervliet & Raes, 2013). Specifically, meta-analyses have shown that, compared to people without anxiety disorders, individuals with anxiety disorders are more likely to fear cues that are safe (i.e. never paired with aversive outcomes), and show problems reducing (extinguishing) their conditional fear responses (Duits et al., 2015). Further preliminary evidence for predictive validity, or the sensitivity of fear conditioning to known disorder treatments, derives from studies showing that pre-treatment responses during the extinction phase of fear conditioning predicts post-treatment outcome in anxious children (Geller et al., 2019; Waters & Pine, 2016), and adults with spider phobia (Forcadell et al., 2017). This task also provides insight into the mechanisms associated with pathological anxiety, in that these differences may reflect a general deficit in inhibitory learning associated with anxiety disorders (Vervliet, Craske, & Hermans, 2013).

There is some evidence to suggest that the acquisition and extinction of fear is moderately heritable (Hettema, Annas, Neale, Kendler, & Fredrikson, 2003), and that individual differences during acquisition, generalisation and extinction of fear are somewhat stable over time (Fredrikson, Annas, Georgiades, Hursti, & Tersman, 1993; Torrents-Rodas et al., 2014). Whilst fear learning and extinction are likely to be influenced by other situational factors, such as stimuli type and presentation context (Torrents-Rodas et al., 2014), these trait-like features indicate that exploration of individual differences in these processes is warranted. As yet we know little about the psychological or biological mechanisms through which these factors operate. We know even less about how individual differences and specific risk factors contribute to outcomes in the development of and extinction of fear (Pittig, Treanor, LeBeau, & Craske, 2018), as few studies have examined individual differences in anxiety and their association with subjective experience of fear conditioning (Lonsdorf & Merz, 2017). Studies that concurrently examine multiple potential mechanisms by which individual differences might occur, in particular when considering genetic influences on a trait, require large sample sizes to minimise the risk of false positives and inflated effect sizes (Munafò et al., 2017). Currently, the time and cost of recruiting and testing participants in a laboratory limits the plausible sample size in fear conditioning research. Of the 48 studies in the largest meta-analysis of fear conditioning differences between anxiety cases and control participants to date (Duits et al., 2015), only 48% reported a total sample size of greater than 50 participants, and just one single study (<3%) reported a sample size of over 100. A sample size of 100, with 50 participants in each group (case/control) provides only 70% power to detect a medium effect between groups (Cohen's d = 0.5), and 17% power to detect a small effect (Cohen's d = 0.2) (Faul, Erdfelder, Lang, & Buchner, 2007). The ability to flexibly and cheaply deliver a task within a range of settings will be needed to detect population level individual differences in associations between fear conditioning and treatment response, or complex interactions between multiple variables. This approach promises to aid in the stratification of risk and prediction of outcomes. In addition, investigations of the genetic underpinnings of the task will require many thousands of participants to achieve sufficient power for the discovery of associated genetic variants (Hong & Park, 2012). Large sample sizes could be achieved if costs of data collection were reduced (Allison, 1997).

One solution is to use smartphone applications (apps) to administer the paradigm. This reduces equipment cost and experiment time and enables rapid data collection from multiple participants simultaneously regardless of location. The Fear Learning and Anxiety Response (FLARe) app was developed to realise this potential. This app administers a fear conditioning paradigm to individuals remotely, without experimenter presence. The studies presented here examined the reliability and validity of this novel app approach by 1) directly comparing performance between app and laboratory administration, 2) assessing within-person test-retest reliability across time and mode of administration, and 3) testing construct validity, or disease relevance, by assessing associations with self-reported anxiety.

## 2 Methods

Three studies were undertaken to investigate the validity and reliability of the FLARe app. First, the cross-modal validation study compared within-person fear conditioning using the app versus traditional laboratory administration. This was the primary study of interest, enabling the assessment of task reliability across mode of delivery. The second and third studies assessed within-person test-retest reliability of laboratory administered, and app administered fear conditioning respectively.

### 2.1 Participants

Participants were volunteers aged 21–26, not pregnant and with no reported history of seizures, neurological or cardiac disorder with access to an Android or iOS smartphone. One hundred participants took part in the validation study of whom eighty-four completed both the laboratory and app delivered paradigm. Fifteen participants (~18%) were excluded due to indicators of poor experimental engagement (non-completion of all phases, reducing phone volume below 70% of the maximum during the acquisition phase, exiting the app, were not contingency aware for one or both testing sessions, or if they did not find the aversive stimulus unpleasant), leaving sixty-nine in the analysis group. The sample size was determined based on 80% power to detect a correlation between fear conditioning variables and anxiety of 0.35 or higher, as associations below this threshold would lead to the conclusion that the smartphone app was not able to detect meaningful individual differences. An additional fifty-one and fifty participants took part in laboratory and app test-retest reliability studies respectively. Of these, forty-seven and fifty individuals respectively completed both sessions, and forty-one and forty-two were included in analyses after excluding for indicators of poor experimental engagement. The sample sizes of these studies were determined based on 80% power to detect between session correlation of 0.45 or greater, as associations substantially below this would lead us to conclude that the task does not capture stable measures of fear conditioning.

This study was performed in accordance with the Psychiatry, Nursing and Midwifery Research Ethics Subcommittees (PNM RESC) of King's College London. (PNM-RES Reference Number: HR15/162349).

### 2.2 Procedure

**Fig. 1:**
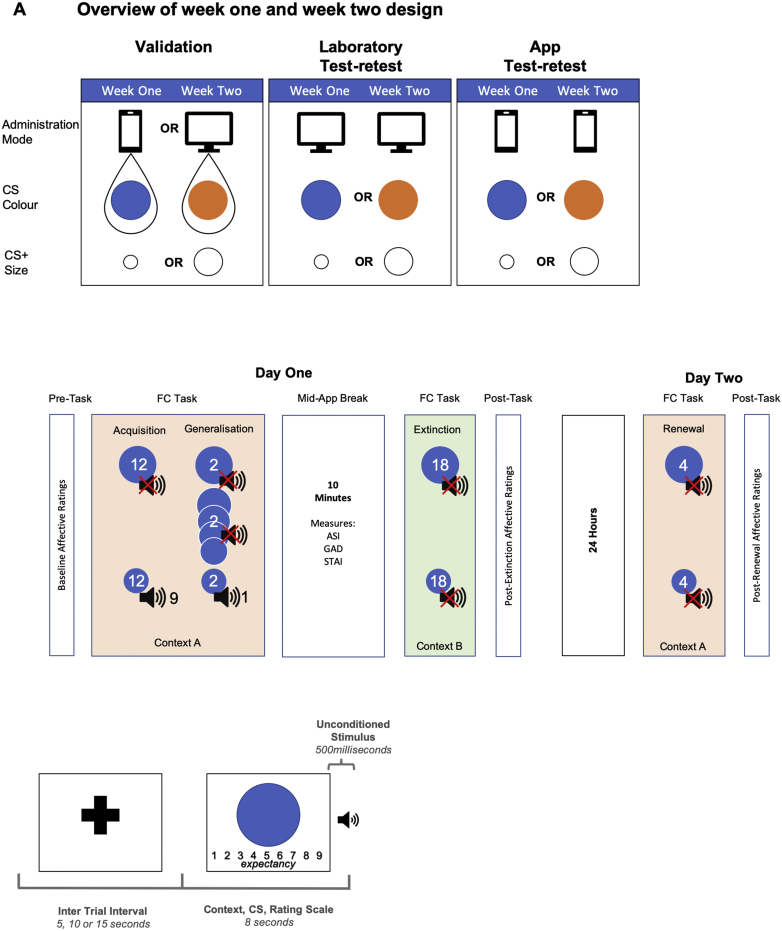
Visualisation of Experimental Design for all studies.

Participants underwent a two-day fear conditioning procedure twice, each consisting of four phases: fear acquisition, generalisation, extinction (day one) and renewal (day two), with a minimum of seven days between the two deliveries. Stimuli were large and small orange or blue circles. Stimuli colour were changed between weeks one and two to ensure conditioning took place in response to different stimuli on the second administration. This has been shown to improve task reliability across time (Torrents-Rodas et al., 2014). The size of the stimulus used as the CS+ was counterbalanced between participants such that approximately 25% of the sample were allocated the smallest circle as a CS+ both weeks, approximately 25% were allocated the largest circle as the CS+ both weeks and approximately 50% of the sample were allocated the largest circle as a CS+ for the first or second week and the smallest circle as the CS+ the remaining week. See Fig. 1, **panel A** for an overview of the three studies, and **panel B** for an overview of the task phases, including detailed counterbalancing. During fear acquisition, participants viewed twelve presentations each of a large and small circle on a background image of an outdoor scene (Context A). A loud (~100 db during laboratory presentation and phone maximum volume for app presentations) human female scream served as the unconditional stimulus (US) and was paired with 75% of the presentations of one out of two circles serving as the conditional stimuli (CS). The circle paired with the scream was counterbalanced between participants and became the CS+ while the circle never paired became the CS-. During generalisation, the CS+ and CS- and another four circles (generalisation stimuli 1–4) were presented two times against Context A. Generalisation stimuli graduated in size between the CS+ and CS-. The smallest generalisation stimulus was 15% larger than the smallest CS, with each subsequent circle being 15% larger again. During generalisation, one presentation of the CS+ was paired with the aversive stimulus. Fear extinction consisted of eighteen presentations each of the CS+ and CS- on a background image of an indoor living room scene (Context B) with no US. Fear renewal involved four presentations each of the CS+ and CS- on Context A with no US.

A minimum break of 10 min occurred between the generalisation and extinction phases, during which participants completed the Spielberger Trait Anxiety Index (Spielberger, 1983), the Generalised Anxiety Disorder 7-item version (GAD-7) (Spitzer, Kroenke, Williams, & Löwe, 2006) and Anxiety Sensitivity Index (Peterson & Heilbronner, 1987) to obtain measures of trait anxiety, current anxiety symptoms and anxiety related cognitions respectively.

### 2.3 Dependent measures

***US-Expectancy ratings.*** For every trial, during each CS/GS presentation, participants recorded how much they expected the stimulus to be followed by a scream (Likert scale *1: certain no scream, 5: uncertain, 9: certain scream*). The scale was available for the last six out of total 8 s of stimulus presentation. Participants’ first response was recorded.

***Affective ratings.*** Participants rated how each stimulus made them feel before the experiment began (baseline), after extinction (post-extinction), and after renewal (post-renewal) on three likert scales. These were “unpleasant” (*1: happy/pleased/content to 9: unhappy/annoyed/despairing)*, “anxious” (*1: calm/sleepy/dull to 9: anxious, aroused, jittery*) and “fearful” (*1: unafraid, safe, unconcerned to 9: fearful, afraid*). Affective ratings were not collected post-acquisition to avoid confounding the early extinction response.

See supplementary information methods for more detail on methods and sFigs. 1 and 2 for details on task instructions.

### 2.4 Data processing

For each study, first mean US-expectancy rating scores were calculated for all stimuli across each phase. See supplementary results, sTable 1-2 for summaries of participants with missing values. Second, a mean of the three affective ratings was calculated to create a negative affect score for baseline, post-extinction and post-renewal due to the significant positive correlation between the different rating types (see supplementary results and sFig. 3) and to reduce measurement error. Finally, to reduce the burden of multiple testing and measurement error a composite anxiety index was created using the average of the normalised total for three significantly correlated anxiety measures; trait (Spielberger, 1983) and general anxiety symptoms (Spitzer et al., 2006) and anxiety sensitivity (Peterson & Heilbronner, 1987). High scores on this composite scale can be thought of as representing higher general domain anxiety across trait, symptom and cognitive domains. See supplementary information for more detail on individual scales, sTable 3 for descriptive statistics and sFig. 4 for intercorrelations between the measures.

### 2.5 Statistical analysis

Repeated measures two-way ANOVAs were used to assess whether mean US-expectancy and affective ratings differed between stimuli during all phases for laboratory and app separately, and between laboratory or app administration for any stimulus/phase in the validation study. Nagelkerke R^2^ were computed comparing the full models including stimulus type, mode of delivery and the interaction between stimulus type and mode of delivery as predictors with participant as a random effect to 1) a null model including only participant as a predictor to establish the variance explained (R^2^) in outcome overall, and 2) a variable only model containing stimulus type and participant as predictors to assess the degree of additional variance in outcome explained by changing mode of delivery. Paired sample t-tests were performed post hoc to examine pairwise differences between stimuli. Next, we tested the consistency of individual performance across different modalities across time relative to the same mode of administration across time. Two-way absolute agreement within-person intraclass correlations (McGraw & Wong, 1996b; 1996a) were computed between weeks one and two for each stimulus/phase, in each study. Intraclass correlations were subsequently transformed to z-scores using Fisher's r to z transformation. Z-tests of the difference between transformed correlations divided by the standard error of the difference (Cohen, Cohen, West, & Aiken, 2003) were performed comparing the validation to the laboratory and app test-retest studies respectively to assess whether the magnitude of intraclass correlations across mode of delivery and across time (validation) differed significantly from those across time alone (laboratory and app test-retest).

Finally, Pearson's correlations were conducted to measure the association between fear conditioning variables and anxiety in laboratory and app data separately. For these analyses we first created two new datasets consisting only of data from the laboratory or app session of the validation study respectively and the first session of the laboratory or app test-retest studies. We correlated each of these fear conditioning measures with composite anxiety. The threshold for statistical significance (p_adj_) was established using matrix decomposition correcting for the effective number of independent tests (m*eff*) after adjusting for intercorrelation of variables for US-expectancy and affective ratings separately (Derringer, 2018). This is similar to undertaking a Bonferroni correction accounting only for the number of truly independent tests.

All analyses were performed using R version 3.5.1 (R Foundation for Statistical Computing, 2017).

## 3 Results

### 3.1 Preliminary analyses

**Fig. 2:**
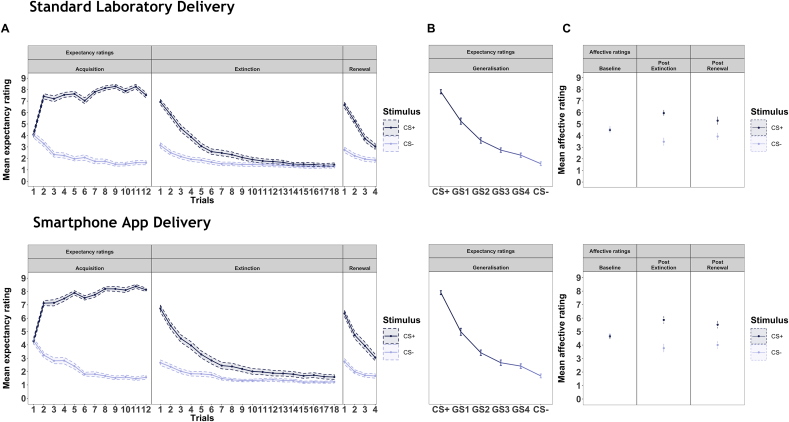
Validation study mean ratings by trial for all stimuli and study phases.

Average US-expectancy and affective ratings for each trial are presented as a function of stimulus, phase and mode of administration for the cross-modal validation study in Fig. 2. Patterns of US-expectancy and affective ratings across stimuli and trials did not differ by mode of administration.

**Fig. 3:**
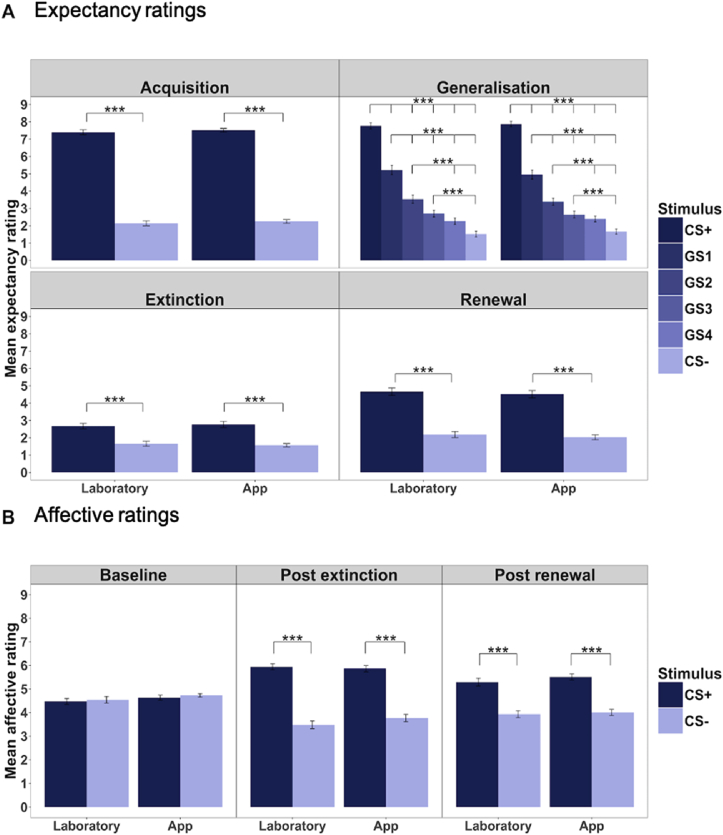
Validation study mean expectancy ratings per phase and stimulus for each mode of delivery.

Results from the ANOVA found a significant main effect of stimulus type for expectancy ratings for all phases, but no significant main effect of mode of delivery or interaction between stimulus and mode of delivery (see Table 1 for results and sFig. 5 in supplementary results for interaction plot showing generalization stimuli). There was no significant main effect of either stimulus type or mode of delivery during the baseline affective ratings, but a significant main effect of stimulus only during post extinction and post renewal ratings (see Table 2). Post hoc testing confirms mean US-expectancy ratings significantly differed between the CS+ and CS- during all experimental phases for both laboratory and app delivery with the largest difference between the CS+ and CS- in acquisition (t _(68)_ = 33.62, t _(68)*=*_ 26.86; see sTable 4 in the supplementary), but did not differ ***between*** laboratory and app administration for any stimulus/phase (As shown in Fig. 3, **panel A**). Further, mean affective ratings differed significantly *between stimuli* for both laboratory and app for all phases except baseline, but did not significantly differ *between modes* of administration for any stimulus during any phase (Fig. 3, **panel B**). Thus, there were no differences between data from the app or laboratory administration of the task averaged across participants for any stimulus or phase in the validation experiment.

**Table 1:** Results of two-way repeated measure ANOVA for validation study US-expectancy ratings.

	Acquisition			Generalisation			Extinction			Renewal		
	DF	*F*	p-value	DF	*F*	p-value	DF	*F*	P-value	DF	*F*	p-value
Intercept	1	9049.51	**<0.0001**	1	914.22	**<0.0001**	1	345.39	**<0.0001**	1	715.81	**<0.0001**
Stimulus	1	939.47	**<0.0001**	5	273.26	**<0.0001**	1	78.54	**<0.0001**	1	168.60	**<0.0001**
Mode of delivery	1	0.47	0.49	1	0.04	0.83	1	0.00	0.99	1	1.44	0.23
Variable by Mode of delivery	1	3.19	0.08	5	0.19	0.96	1	0.14	0.71	1	0.31	0.58

Nagelkerke R^2^ Full vs Null	0.90			0.67			0.28			0.43		
Nagelkerke R^2^ Full vs Stimulus only	0.13			0.06			0.006			0.02		

Table showing the results for each phase of the validation study of two-way repeated measures ANOVA with stimulus type, mode of delivery and stimulus type by mode of delivery interaction as fixed effect predictors of mean US-expectancy ratings. US-expectancy rating for each stimulus averaged across all trials of each phase for laboratory and app administration for the remote validation study (n = 69). Stimuli for the Acquisition, Extinction and Renewal phases include the CS+ and CS-. Stimuli for the Generalisation phase include the CS+, CS- and the four generalisation stimuli (GS1,2,3 and 4). Modes of delivery include app and laboratory administered a week apart in all cases. p-value of significant predictors are emphasised in **bold**.

CS+; the conditional stimulus that is paired with the aversive sound during acquisition and generalisation.

CS-; the conditional stimulus that is never paired with an aversive sound.

GS1-GS4; Generalisation stimuli ranging from the most to least similar in appearance to the CS+.

Nagelkerke R^2^ Full vs Null; Pseudo R^2^ value derived by comparing the variance explained by the full model to a null model with only participant included as a random effect.

Nagelkerke R^2^ Full vs Stimulus only; Pseudo R^2^ value derived by comparing the variance explained by the full model to a model with only the fixed effect of stimulus included as a predictor. Thus this value represents the additional variance explained when including mode of delivery as a predictor.

**Table 2:** Results of two-way repeated measure ANOVA for validation study affective ratings.

	Baseline			Post extinction			Post renewal		
	DF	*F*	P	DF	*F*	P	DF	*F*	P
Intercept	1	3580.84	**<0.0001**	1	2223.65	**<0.0001**	1	1801.39	**<0.0001**
Stimulus	1	0.45	0.49	1	217.14	**<0.0001**	1	52.71	**<0.0001**
Mode of delivery	1	3.40	0.06	1	0.84	0.36	1	3.78	0.05
Variable by Mode of delivery	1	0.33	0.56	1	2.33	0.13	1	1.05	0.31

Nagelkerke R^2^ Full vs Null	0.05			0.47			0.29		
Nagelkerke R^2^ Full vs Stimulus only	0.04			0.01			0.06		

Table showing the results for each phase of the validation study of two-way repeated measures ANOVA with stimulus type, mode of delivery and stimulus type by mode of delivery interaction as predictors of mean affective ratings. Remote validation study (n = 69). Stimuli for all phases include the CS+ and CS-. Modes of delivery include app and laboratory administered a week apart in all cases. p-value of significant predictors are emphasised in **bold**.

Affective ratings; Composite affective rating comprising of self-reported feelings of anxiety, fear and unpleasantness for each stimulus at three time points i) before the experiment begins (baseline), after the extinction phase (post-extinction) and after day two renewal (post-renewal).

CS+; the conditional stimulus that is paired with the aversive sound during acquisition and generalisation.

CS-; the conditional stimulus that is never paired with an aversive sound.

Nagelkerke R^2^ Full vs Null; Pseudo R^2^ value derived by comparing the variance explained by the full model to a null model with only participant included as a random effect.

Nagelkerke R^2^ Full vs Stimulus only; Pseudo R^2^ value derived by comparing the variance explained by the full model to a model with only the fixed effect of stimulus included as a predictor. Thus this value represents the additional variance explained when including mode of delivery as a predictor.

### 3.2 Cross-modal validation

**Fig. 4:**
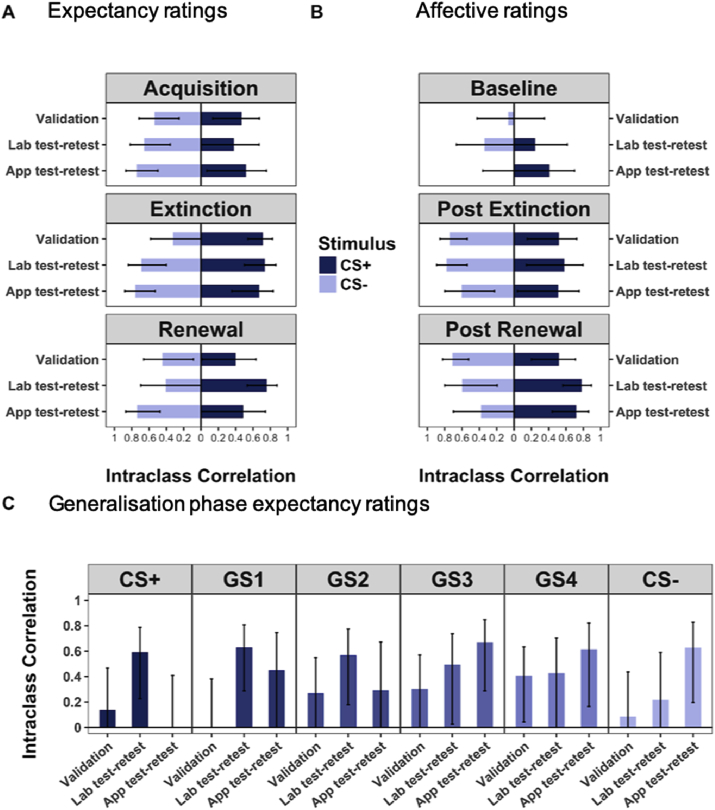
Absolute agreement intraclass correlations between overall stimulus means across testing sessions.

Fig. 4 shows the two-way absolute agreement intraclass correlations for all stimuli for all phases for the validation, laboratory test-retest and app test-retest studies. Intraclass correlation estimates for the **validation study**, comparing within-person correlations for the experiment in the laboratory vs the app a week apart were largest for mean US-expectancy ratings for the CS+ during extinction (ICC = 0.72, ci_95%_[0.54–0.82]) and CS- during acquisition (ICC = 0.54, ci_95%_[0.25–0.71]) where they were moderate to strong (Koo & Li, 2016). See sTable 5 in the supplementary for intraclass correlation for all studies. Magnitude of the intraclass correlations did not differ between the validation study and either the laboratory or app test-retest studies for any stimuli for any phase or rating type. Thus, there were no differences in individual task performance by mode of administration. There were no differences in perceived US unpleasantness between app and laboratory delivery (see supplementary results, sTable 6 for details).

### 3.3 Associations with anxiety

#### 3.3.1 Laboratory data

**Fig. 5:**
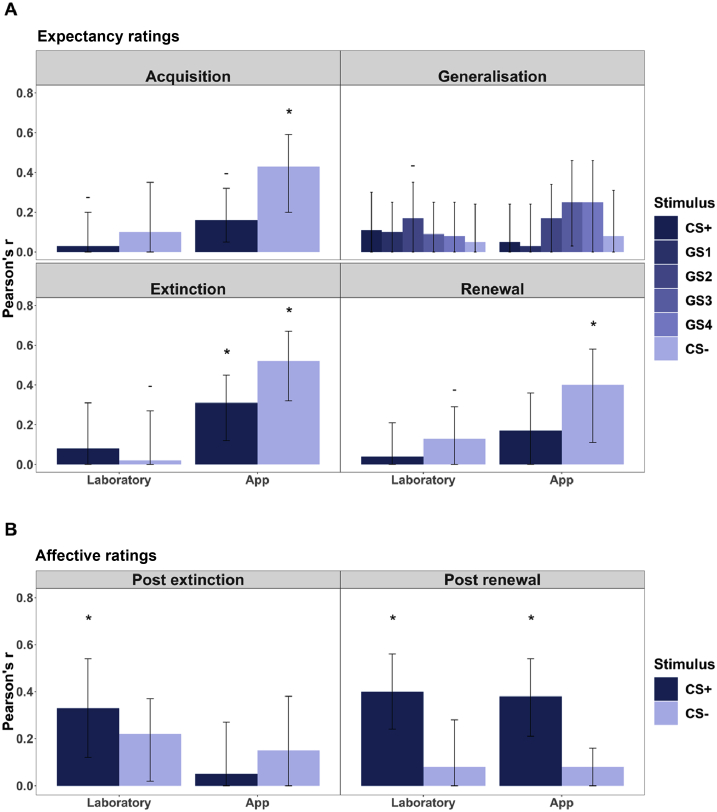
Correlations between composite anxiety score and fear conditioning variables administered by standard laboratory or smartphone app.

See Fig. 5 for correlations between anxiety and fear conditioning variables. Correlations between composite anxiety and US-expectancy ratings for either CS were not significant during laboratory administration of the task, after correcting for the effective number of independent tests (m*eff* = 10.56, p_adj_ < 0.005). Mean affective ratings of the CS+ were significantly correlated with composite anxiety at both post-extinction (r = 0.33, p = 0.004) and post-renewal (r = 0.4, p < 0.001) time points after correcting for the effective number of independent tests (m*eff* = 3.6, p_adj_ < 0.01).

#### 3.3.2 App data

Correlation between anxiety symptoms and mean US-expectancy ratings to the CS+ was significant during the extinction phase (r = 0.31, p = 0.004). Correlations between composite anxiety and mean US-expectancy ratings for the CS- during the acquisition (r = 0.43, p < 0.001) and extinction phase (r = 0.52, p < 0.001) were significant after correcting for the effective number of tests (m*eff* = 10.56, p_adj_ < 0.005). Mean affective ratings of the CS+ post-renewal was significantly correlated with composite anxiety scores (r = 0.38, p < 0.001) after correcting for the effective number of independent tests (m*eff* = 3.6, p_adj_ < 0.01).

To investigate the stability and validity of stimulus discrimination all analyses were repeated using the differential between the CS+ and CS- for all phases. See supplementary results sTable 7 and sFig. 6 for details of these sensitivity analyses. Sensitivity analyses were performed to assess the correlation with outcome measures and the individual anxiety measures used to create the composite. See supplementary results sFig. 7.

## 4 Discussion

This study evaluated the validity of a smartphone app remotely-delivered differential fear conditioning task. First, we showed that patterns of trial-by-trial responding did not differ when the task was administered using the newly developed FLARe app or in a laboratory setting. Second, we demonstrated high within-person cross-delivery-mode correlations for fear learning. Finally, we showed that individual differences in fear conditioning outcomes were associated with anxiety, providing construct validity in that the task shows relevance to the disorder of anxiety. Further, by identifying those who have higher overall anxiety, the app task demonstrates some level of diagnostic validity (Vervliet & Raes, 2013). Below we discuss these three sources of validation evidence.

### 4.1 Patterns of differential fear conditioning

Participants displayed differential conditioning regardless of mode of administration, with significantly higher average US-Expectancy ratings for the CS+ than the CS- by the end of the fear acquisition phase. Similarly, for both modes of delivery, participants “generalised” their fear to related stimuli, reduced their expectation of aversive outcomes over the course of extinction, and demonstrated renewed US-expectancy on day two. Average ratings of negative affect were greater to the CS+ than the CS- after the extinction and renewal phases regardless of administration mode. Together, the results show that the app produced a pattern of differential conditioning, extinction and renewal that was very similar to the pattern observed in the laboratory setting. We found that cross-modal reliability assessed by within-person intraclass correlations was comparable to reliability across time when mode of administration remained constant. This indicates that test-retest reliability was not impacted by mode of administration.

### 4.2 Cross-modal within-person validation

Intraclass validation correlations were moderate (between 0.5 and .75) (Koo & Li, 2016) for most phases and stimuli. They were below 0.5 for CS- US-expectancy ratings during extinction, for all stimuli during the generalisation phase, and both the CS+ and CS- baseline affective ratings. Of note, it is possible that fear conditioning differs when undertaken a second time, owing to residual learning from the first occasion. Although we altered stimuli colour (blue versus orange) at testing in line with findings by Torrents-Rodas et al. (2014) that test-retest reliability is substantially greater when the stimuli are varied across testing session. The fact these correlations are <1.0 is unsurprising and is in keeping with previous test-retest studies of US-expectancy ratings (Torrents-Rodas et al., 2014). We note that any residual learning effects such as spontaneous recovery, poor forgetting or impaired discriminatory learning will result in lower test-retest reliability across time alone. Thus, it is likely that what we present represents the lower bound of the app validity (which is the stability of performance across mode as well as across time). The low validation correlations for CS- US-expectancy ratings during extinction, all stimuli during generalisation, and affective ratings at baseline warrant further consideration. Whilst these low correlations may reflect high within-person variance (Bravo & Potvin, 1991) (i.e. low agreement across time/delivery mode), they can also result from low between-person variance (i.e. little variation in the measure of interest within the group). US-expectancy ratings for the CS- during extinction were consistently low for all participants/trials, leading to low between-person variation in responses. In contrast, generalisation was assessed using only two trials per stimulus, so within-person variance may have been unduly influenced by extreme ratings at either time point. Another possible explanation for low intraclass correlation for generalisation stimuli is that after the first testing session participants learn that changing size dimension does not cue a US, and thus perform differently during the second administration of the task. Baseline affective ratings the second time participants engage in the task are likely to be influenced by previous learning experiences. Thus, low intraclass correlations here were likely due to larger within-person variation. Despite this, baseline ratings remain useful variables to control for any pre-existing biases towards the neutral stimuli.

### 4.3 Associations with anxiety

Anxiety was associated with *higher* US-expectancy to the CS- during the acquisition phase, and *higher* US-expectancy to both the CS+ and CS- in app administration only. Significant associations were seen between post-task negative affect toward the CS+ and anxiety in both laboratory and app administrations. These findings echo those from a meta-analysis of fear conditioning studies, which found that “over-generalising” fear responding and reduced or delayed extinction of fear responding distinguished cases from controls across a range of anxiety disorders (Duits et al., 2015). Of note, in our non-clinical sample, this association was only evident when the experiment was administered via the app. Previous studies have largely failed to find any associations between individual differences in anxiety and subjective fear conditioning ratings using laboratory procedures (Lonsdorf & Merz, 2017). This might be due to between-person variation differing as a function of the degree of control over the testing situation. Experiments designed to elicit individual differences on the whole benefit from greater between-participant variation (Hedge, Powell, & Sumner, 2018). Laboratory administered fear conditioning, with researcher guidance and consistent environment, may produce a *strong situation* where the task is experienced in virtually the same way by all, minimising between-person variation and reducing detection of individual differences (Lonsdorf et al., 2017). The ambiguity of the task under app administration conditions with no researcher presence and inconsistent environment might encourage a *weak situation* (Lissek, Pine, & Grillon, 2006), allowing for greater inter-individual variation*.* Previous studies that have identified associations between fear conditioning and individual differences under situations of ambiguity (Lonsdorf & Merz, 2017; Staples-Bradley, Treanor, & Craske, 2018; Wong & Lovibond, 2018) support this supposition. Thus, the app may be better suited to detecting individual differences in US-expectancy ratings. Future investigations may be able to test this hypothesis.

Negative affect was significantly associated with anxiety in both modes of delivery. Our approach of capturing overall negative affect towards the stimuli has not previously been considered, and it is possible that the additional variance in these responses might make them better suited for detecting individual specific variation in laboratory situations.

### 4.4 General limitations

The FLARe app and our comparison laboratory procedure used an aversive human scream sound as the unconditional stimulus rather than an electrodermal shock. Although results from several studies find that fear learning occurs equally well when reinforced by a scream (Glenn, Lieberman, & Hajcak, 2012; Lau et al., 2008), there is some evidence that a shock results in a larger magnitude of startle response and is potentially more sensitive to individual differences (Glenn et al., 2012; Lau et al., 2008). Thus, our app was not validated against what many consider to be the field ‘gold standard’. Although this presents a potential limitation to the generalisability of the FLARe app, it does make the experiment more suitable for work in younger populations (Neumann, Waters, & Westbury, 2008).

There are some additional limitations to the outcome measures used. The app task is currently only able to collect self-report measures of anticipatory and evaluative fear (US-expectancy ratings and affective ratings respectively). These can be thought to represent the verbal components of fear, but not the physiological or behavioural components (Lang, 1971). Although the correspondence between these and biophysiological measures of fear is not clear (Lipp, 2006) self-report ratings demonstrate face, construct, diagnostic and predictive validity (Boddez et al., 2013). Future developments of the app would benefit from the inclusion of biophysiological outcome measures. Further, the use of a single anxiety and negative affective rating composite, whilst reducing measurement error and the burden of multiple testing, loses specificity which we recognise is a limitation.

For the comparison of intraclass correlations between the validation and laboratory and app test-retest studies post hoc power analyses given our respective study sample sizes suggest that we drop below 80% to detect differences in correlations between our studies with an effect size (q) of 0.52 or smaller. To put this into perspective, if the correlations differ in magnitude by <~ r 0.3 we would unlikely be powered sufficiently to detect this. Thus, it is possible that there are differences in the intraclass correlations that exceed this threshold and remain undetected. Intraclass correlations may have been influenced by the decision to vary stimulus by size randomly on both occasions. This resulted in approximately half of the sample being allocated the same size circle as the CS+ for both administrations, while the other half received a different size circle. This additional variability in session two may have reduced the intraclass correlations detected in this study.

### 4.5 Future directions

Whilst the fear learning results were comparable across both forms of delivery, it will be important to assess any technical differences between these methods, for example with regards to drop-out and participation rates. The FLARe app also requires further validation in patient populations or clinical settings. The FLARe app is currently being developed as a flexible research tool to enable future collaborative research efforts across a range of study designs, settings, and populations. This validation presents preparatory work for use of the FLARe app in large samples, including the Twins Early Development Study (TEDS).

## 5 Conclusions

Our analyses have demonstrated that smartphone delivery of a fear conditioning paradigm resulted in a pattern of fear learning comparable to traditional laboratory delivery and was able to detect individual differences in fear learning associated with anxiety. The use of smartphone technology for data collection will enable the acquisition of substantially larger samples than is currently feasible. This in turn will allow researchers to explore the interactive effects of multiple predictors of anxiety development, maintenance and treatment response.

## Open practices statement

None of the experiments reported in this article were formally preregistered. Neither the data nor the materials have been made available on a permanent third-party archive. Future iterations of the FLARe app will be made open source. Enquiries regarding use of the app or requests for the data or materials can be sent via email to the lead author at kirstin.purves@kcl.ac.uk.
