# Supplementary material for: Validating the use of a smartphone app for remote administration of a fear conditioning paradigm
Source: Behav Res Ther. 2019 Dec;123:103475. doi: 10.1016/j.brat.2019.103475 (PMC6891256; doi:10.1016/j.brat.2019.103475)
Supplement: Multimedia component 1 [file mmc1.pdf]

Supplementary Information

Methods

Participant recruitment

Participants were recruited through a combination of online advertising (via email and participant recruitment websites based at King's College London and Goldsmiths Universities in London, Instagram, Facebook and Twitter online services) and posters placed at various locations in and around South East London, UK.

Participants were restricted to individuals with access to iOS or Android smartphones. The validation study was conducted first and thus any participants who made contact with the team for the duration of this study were allocated to the Validation study if eligible and were able to access the test centre based in South East London.

The app and laboratory test-retest studies were conducted simultaneously. Individuals were assigned to the app test-retest study if they were outside of the area of south-east London, and to the lab test-retest if they were within reach of the testing centre.

Procedure

See **sFigure 1** and **sFigure 2** for screenshots showing set up and task instructions respectively. Prior to beginning the experiment, participants were shown images of the CS+ and CS- and asked to rate each for familiarity (How uncertain/certain they are that they have seen the image before), valence (How happy/unhappy does the image make them feel?), arousal (how calm/anxious does the image make them feel?) and fear (How unafraid/afraid does the image make them feel?) on a scale of 1 – 9.

During the experimental phases, stimuli were presented in a pseudo-random order, with the following conditions: (a) the first two trials were a CS+ and CS-, (b) the first occurrence of the CS+ was always reinforced with the US and (c) no stimulus was repeated for more than two consecutive trials.

Each trial lasted for a total of 8 seconds. Online expectancy ratings were obtained for every trial. After 2 seconds, a rating scale appeared on screen asking the participants to rate how much they expect the US to occur (1 – completely certain no US will occur, 9 – completely certain US will occur). During laboratory administration responses were made using a standard keyboard. During app administration responses were made using their phone touch screen. During mobile administration, if participants missed two consecutive ratings, they

saw a pop-up notification reminding them to continue making their online expectancy ratings. US onset was at 7.5 seconds and co-terminated with the CS+. Intertrial intervals varied randomly between 5,10 and 15 seconds.

During acquisition, participants were shown 12 presentations each of the CS+ and CS-. The CS+ was reinforced with the US on 75% of trials. During generalisation, each of the 6 total stimuli were shown two times each. The CS+ was reinforced with the US on 50% of the trials. During extinction, the CS+ and CS- were shown 16 times each, with no US reinforcement.

A forced ten-minute break occurred between generalisation and extinction. During this time, participants were redirected to another laptop (lab administration), or external site (mobile administration) to complete questionnaires. First, participant's contingency awareness was assessed: participants were shown the question "Did you happen to notice whether the scream occurred with one of the shapes?", if they indicated they did, they were asked to indicate which circle size the scream had occurred with. If participants responded in the affirmative to question 1, and correctly identified the circle size of their CS+, they were considered contingency aware. Participants were then presented with questionnaires, including the Generalised Anxiety Disorder-7 (GAD-7) (Spitzer, Kroenke, Williams, & Löwe, 2006), Anxiety Sensitivity index (Peterson & Heilbronner, 1987), and the Trait subscale of the State and Trait Anxiety Inventory (STAI – T) (Spielberger, 1983).

After generalisation, during the ten-minute break before extinction, participants were asked to rate how unpleasant they found the US (1 – not at all unpleasant; 9 – highly aversive and unpleasant), and whether they noticed if the scream followed a particular shape, and if so which. This established whether they were aware of the contingency between the scream and the CS+. After extinction, participants were asked to rate all six experimental stimuli for familiarity, valence, arousal and fear for a second time.

Counterbalancing

Validation study

The order of session (laboratory or app) was randomly assigned, such that approximately half of the participants underwent mobile administration first, and half underwent lab administration first. The colour of the circles used as the conditioned stimuli were always blue when the task was administered by app, and orange when administration was in the laboratory. The size of the circle that served as the CS+ was counterbalanced across all participants.

Laboratory and app test-retest.

The colour of the circles used as the conditioned stimuli were counterbalanced across all participants, with the condition that the colour differed between week one and two.

The size of the circle that served as the CS+ was counterbalanced across all participants.

sFigure 1. Screenshots of the FLARe app set up instructions

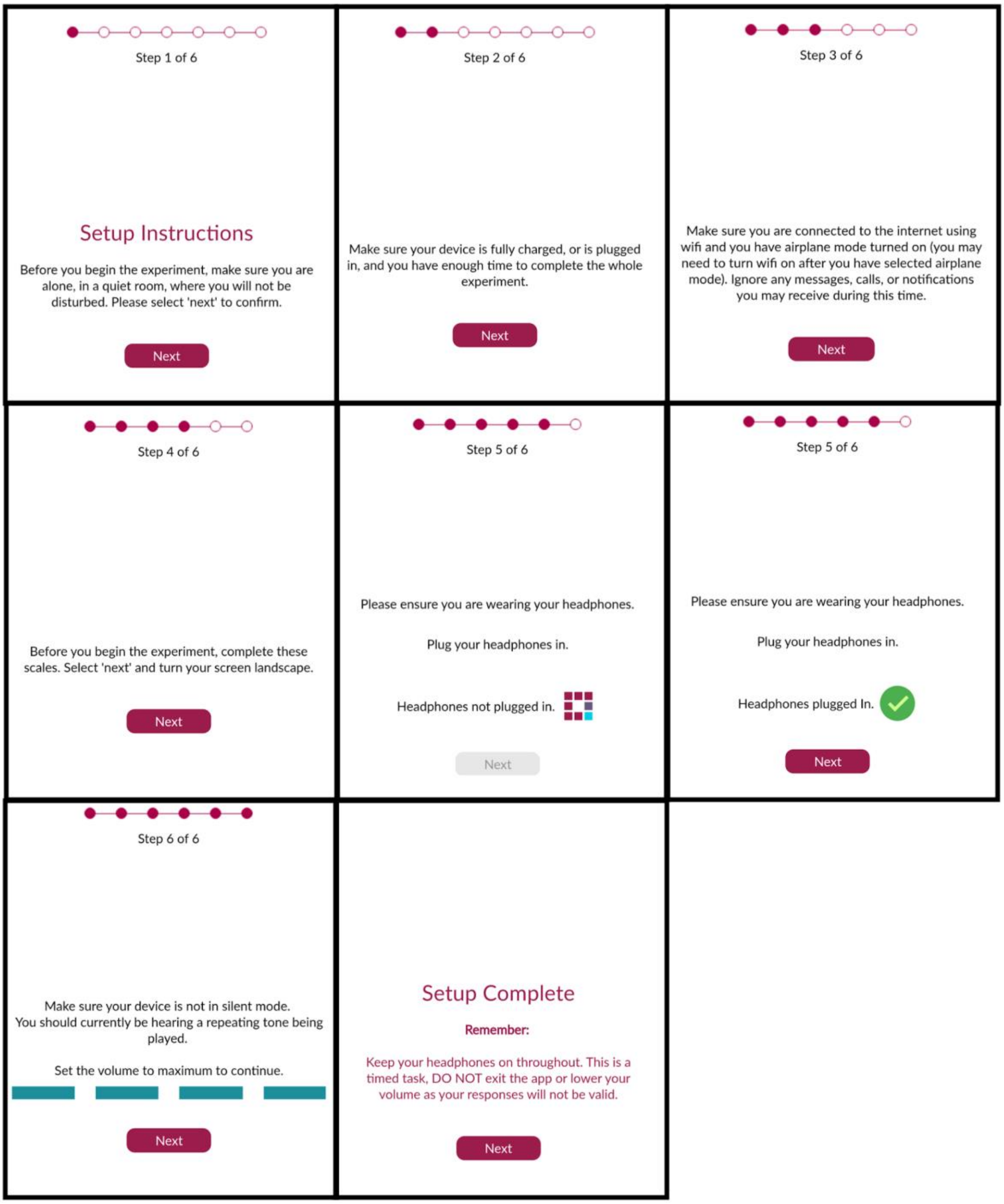

sFigure 1. Figure showing screen shots from the FLARe app set up phase. Users see these instructions after logging into the app and before the fear conditioning task commences. Panels should be read from left to right for each consecutive row.

sFigure 2. Screenshots of the FLARe app task instructions

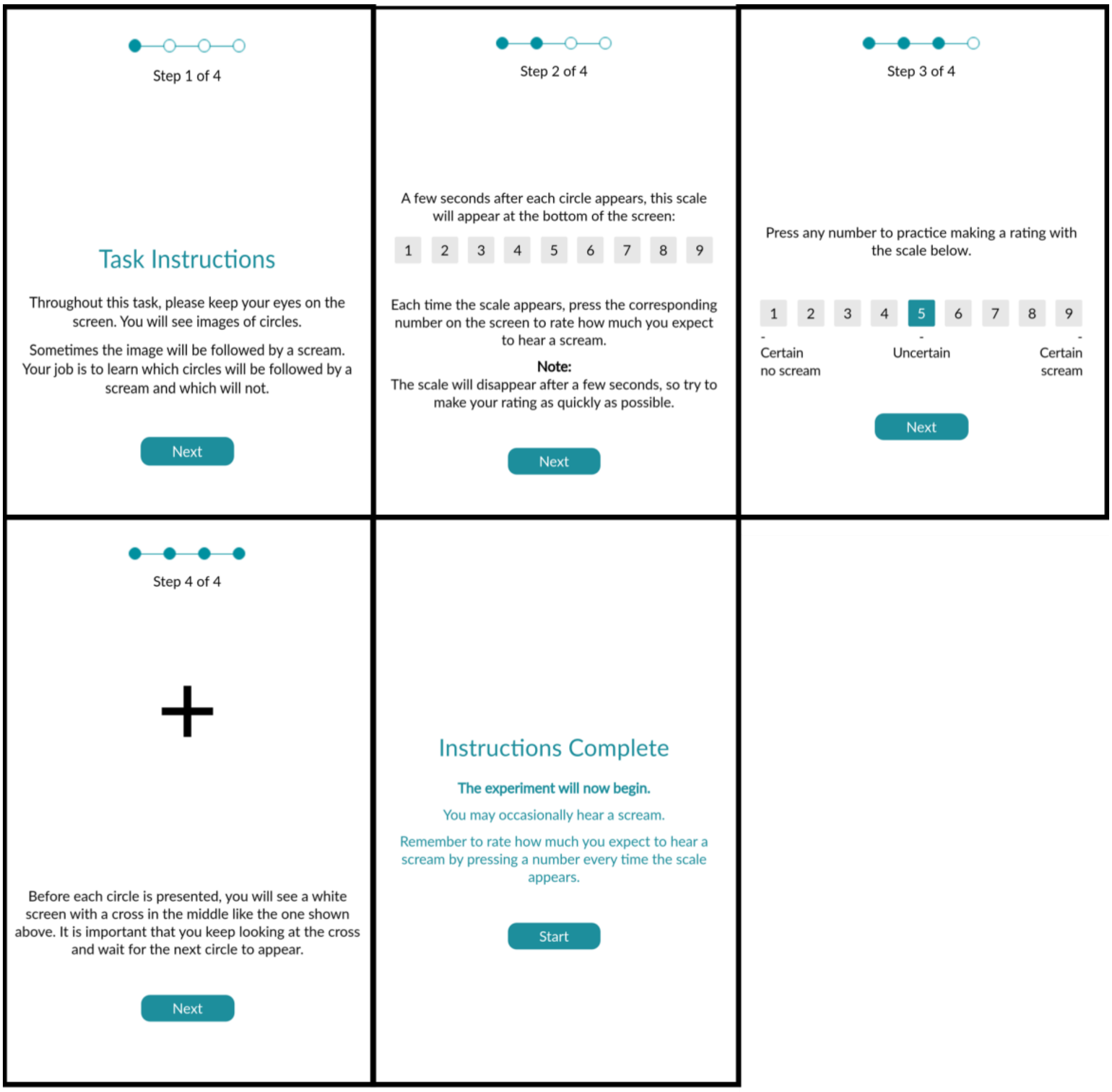

sFigure 2. Figure showing screen shots from the FLARe app experimental instruction phase. Users see these instructions after logging in and going through initial set up guidance (see **sFigure 1**). These instructions immediately precede the beginning of the fear conditioning experiment.

Stimuli

Six circles varying in size served as the CS+, CS- and GS. Each circle increased in size by 15%, in this way the relative size difference between stimuli was maintained despite differences in screen sizes. For the validation study, during lab administration circles were orange. During mobile administration circles were blue. For the lab and app test-retest studies the circle colour at week one and week two was counterbalanced for all participants. During acquisition and generalisation phases, circles were presented on a context image consisting of an outdoors garden scene. During extinction, circles were presented on a context image of an indoor living room scene.

The US was a human scream, played for a duration of 500ms. The scream was played using a set of over-ear headphones. The same headphones were used for both lab and mobile administration. Headphones were provided to all participants prior to the mobile administration. During lab administration, the scream was played at 100db. During mobile administration, the scream was played at the individual phone's maximum volume.

Data processing.

Imputation of missed values for US-expectancy ratings

If the first rating of any given stimulus type during acquisition or extinction phase was missed, the starting value was set as an average of all participants starting values for that stimulus type, administration method and phase.

If the final rating of any given stimulus type during acquisition or extinction phase was missed, the value was set by carrying forward of the last rating made for that stimulus type, administration method and phase

If any rating between the first and last rating for any stimulus was missed during acquisition or extinction, the value was calculated as an average of the preceding and proceeding rating.

Creation of anxiety composite score

Trait anxiety was assessed using each participant's total score on the 20 item trait scale from the Spielberger State-Trait Anxiety Inventory (Spielberger, 1983). Generalised anxiety was assessed using total score from the GAD-7 (Spitzer et al., 2006). Anxiety sensitivity was measured using total scores from the Anxiety Sensitivity Index (Peterson & Heilbronner, 1987). To create the total anxiety composite, z-scores were derived from the total scores from each of the three anxiety measures for laboratory and app task administration data separately. Final composite score was a mean of these three z-scores for each participant.

Exclusion for differing contingency awareness

Participants were excluded if they were not contingency aware for either one or both of the two testing sessions. This resulted in the loss of six participants who were only aware of the contingency between the US and the CS+ during one testing session. No participants were contingency unaware for both sessions. Of these six participants, five were not contingency aware during the first test session but were by the end of session two. The remaining individual was contingency aware for the first, but not second test session.

CS+ / CS- differential analyses

Secondary analyses were performed using the differential between the CS+ and CS- for each rating type for each phase (calculated as subtracting the mean CS- value from the mean CS+ value). This was to investigate the stability and construct validity of the ability of individuals to discriminate between stimuli.

Results

Data processing.

A summary of proportion of participants who missed any values per phase by mode of administration is presented in **sTable 1 and 2** below, in addition to a summary of average and modal number of trials missed per person.

sTable 1. Proportion of participants who missed any trials

	Acquisition		Extinction		Renewal	
	**CS+**	**CS-**	**CS+**	**CS-**	**CS+**	**CS-**
**Laboratory**	13%	18%	27%	18%	5%	5%
**App**	13%	17%	16%	14%	4%	4%

sTable 2. Summary of number of trials missed by phase:

	Acquisition			Extinction			Renewal		
	**Mean**	**Mode**	**Range**	**Mean**	**Mode**	**Range**	**Mean**	**Mode**	**Range**
**Laboratory**	1.31	1	1-4	1.43	1	1-4	1	1	1-1
**App**	1.73	1	1-8	2.04	1	1-9	1	1	1-1

Individual affective ratings

Within both the app and laboratory samples all affective ratings were significantly positively correlated with each other (Laboratory CS+ range r= 0.53 - 0.82, mean r = 0.70, CS- range r= 0.52 - 0.67, mean r = 0.59; App CS+ range r= 0.63 - 0.86, mean r = 0.73, CS- range r= 0.62 - 0.86, mean r = 0.72). See **sFigure 3** for heatmap showing these associations.

sFigure 3. Correlations between individual affective ratings by stimuli type, phase and mode of delivery

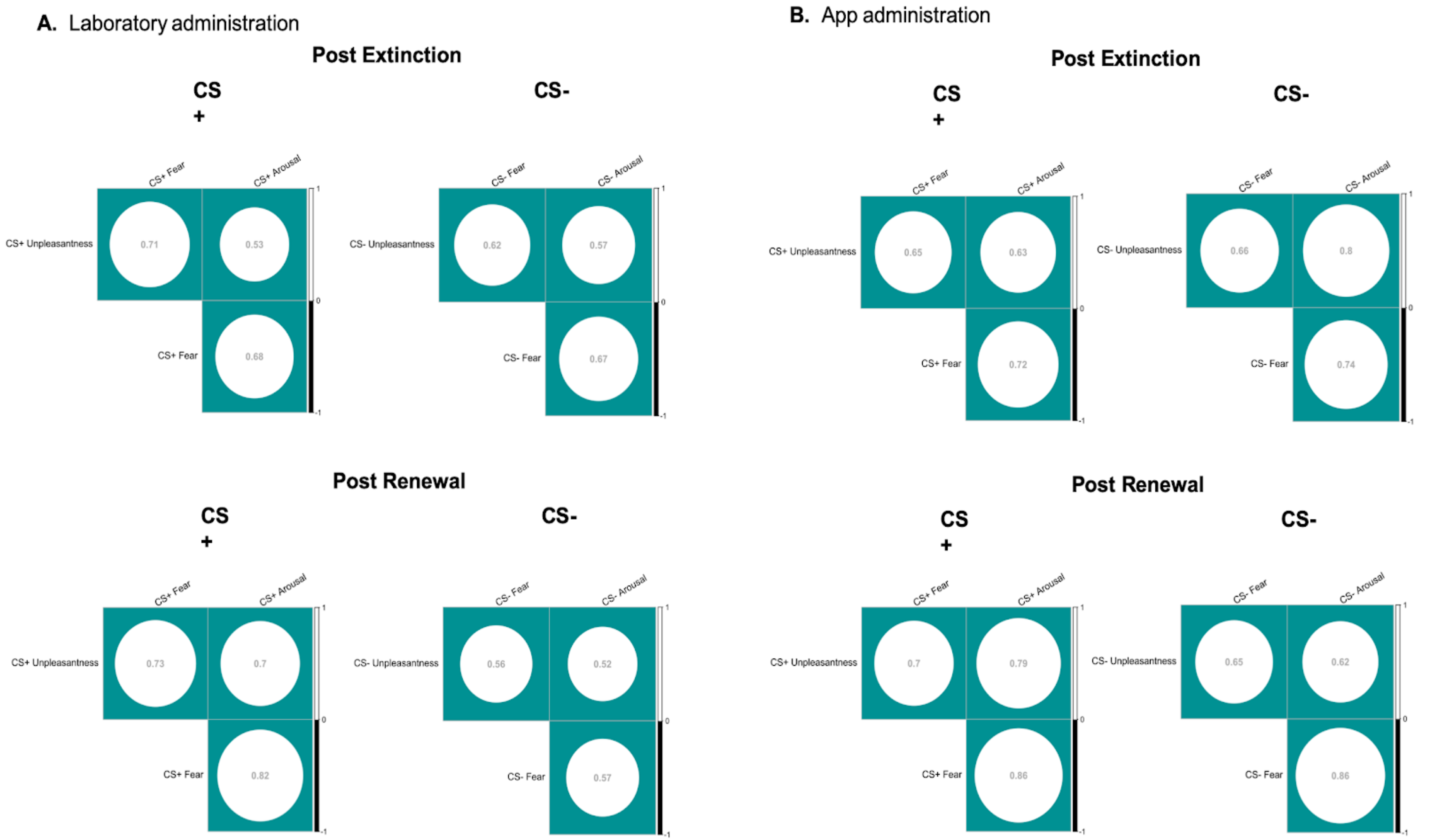

sFigure 3. Heat plots visualising the correlation between the three individual affective ratings (Fear; how afraid does this image make you feel, Arousal; How anxious does this image make you feel?, Valence; How unpleasant does this image make you feel?) all rated on a scale of 1-9, with 1 being the least fearful/anxious/unpleasant and 9 being the most fearful/anxious/unpleasant. Intercorrelations between the affective ratings are shown for each stimulus (CS+, CS-) made after the extinction phases *(Post extinction)* and after the renewal phase *(Post renewal)* for Laboratory *(left)* and App *(right)* testing sessions respectively.

Colour of the circle indicates direction of the association (white = positive, black = negative), size of the circle indicates the strength of the relationship (larger circles indicate higher Pearson's r value).

Non-significant correlations (P > 0.05) are crossed out using a large black 'x' symbol

Individual anxiety measures

Within both the app and laboratory samples all anxiety measures were significantly correlated with each other. See **sTable 3** for a summary of results for each anxiety measure for each study. See **sFigure 4** for a correlation matrix of these associations.

sTable 3. Summary results for each anxiety component by mode of delivery

	Mean	St.Dev	Min	Max
**Laboratory**				
**GAD-7**	5.82	4.78	0.00	18.00
**Trait Anxiety Inventory**	43.45	11.27	22.00	70.00
**Anxiety Sensitivity Index**	22.39	12.11	3.00	60.00
**App**				
**GAD-7**	5.14	4.41	0.00	21.00
**Trait Anxiety Inventory**	42.49	11.30	23.00	76.00
**Anxiety Sensitivity Index**	24.50	11.61	8.00	63.00

Table showing the mean, standard deviation and range of scores on the three anxiety measures that make up the anxiety composite score for combined laboratory and app administration.

GAD-7; Generalised Anxiety Disorder-7,
St. Dev.; Standard deviation of scores

sFigure 4. Correlations between individual anxiety scales in laboratory and app

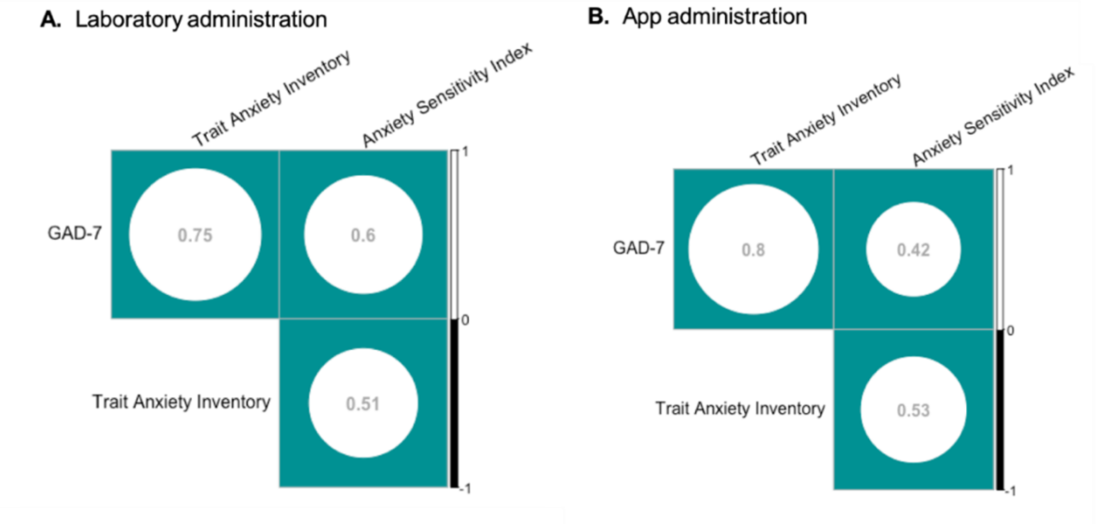

sFigure 4. Plots visualising the correlation between the three individual anxiety scales (Trait anxiety, Generalised Anxiety Disorder-7 symptoms, Anxiety Sensitivity) for first week only in validation, app test-retest or Laboratory test-retest. Correlations presented for the app (n = 89) and Laboratory (n = 91) based testing separately.
Colour of the circle indicates direction of the association (white = positive, black = negative), size of the circle indicates the strength of the relationship (larger circles indicate higher Pearson's r value). Non-significant correlations (P > 0.05) are crossed out using a large black 'x' symbol.

Preliminary analyses

Supplementary tables and figures showing mean expectancy summaries across phases.

sFigure 5. Interaction plot of generalisation stimuli by mode of delivery

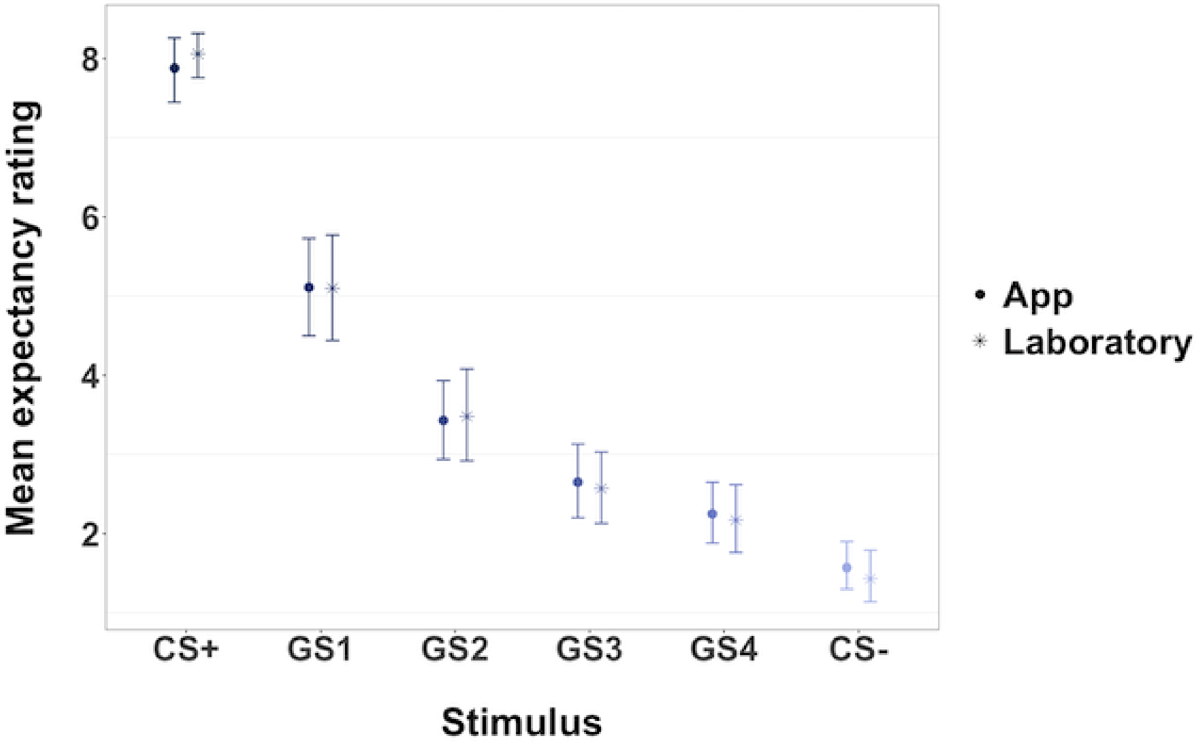

sFigure 5. Interaction plot showing the mean expectancy rating for each stimulus during the generalisation phase of the validation experiment for the laboratory and app mode of delivery respectively.

sTable 4. Mean stimulus US-expectancy for remote validation study

Phase	Mode	Stimulus Mean (standard error)		Difference	t	p-value
		CS +	CS -			
**US-expectancy**						
**Acquisition**	**Laboratory**	7.41 (0.15)	2.14 (0.14)	5.27	33.62	2.2x10-16*
	**App**	7.52 (0.11)	2.52 (0.11)	5.00	26.86	2.2x10-16*
**Generalisation**	**Laboratory**	7.77 (0.16)	1.52 (0.16)	6.25	34.08	2.2x10-16*
	**App**	7.87 (0.15)	1.66 (0.15)	6.21	26.07	2.2x10-16*
**Extinction**	**Laboratory**	2.67 (0.16)	1.66 (0.14)	1.01	7.30	4.0x10-10*
	**App**	2.77 (0.17)	1.57 (0.09)	1.20	7.60	1.3x10-10*
**Renewal**	**Laboratory**	4.66 (0.21)	2.19 (0.17)	2.47	9.50	4.9x10-14*
	**App**	4.52 (0.21)	2.03 (0.14)	2.49	9.56	6.1x10-14*
**Affective ratings**						
**Baseline**	**Laboratory**	4.47 (0.13)	4.54 (0.14)	-0.07	0.75	4.5x10-01
	**App**	4.64 (0.11)	4.73 (0.07)	-0.06	0.13	8.8x10-01
**Post-extinction**	**Laboratory**	5.94 (0.13)	3.48 (0.16)	2.46	11.04	2.3x10-16*
	**App**	5.86 (0.13)	3.77 (0.15)	2.09	8.28	5.3x10-11*
**Post-renewal**	**Laboratory**	5.29 (0.16)	3.92 (0.15)	1.37	5.61	4.5x10-07*
	**App**	5.51 (0.13)	4.00 (0.13)	1.51	7.32	4.9x10-10*

Table showing the mean (standard error) US-expectancy rating for each stimulus averaged across all trials of each phase for laboratory and app administration for the remote validation study (n=69). Shaded portion shows the absolute difference between US-expectancy rating to the CS+ and CS- and results of t-test comparing the mean US-expectancy for the CS+ and CS- for laboratory and app administration respectively. Significant differences are indicated by a "*".

US-expectancy; Average self-reported US-expectancy rating per stimulus across all trials for each phase. Affective ratings; Composite affective rating comprising of self-reported feelings of anxiety, fear and unpleasantness for each stimulus at three time points i) before the experiment begins (baseline), after the extinction phase (post-extinction) and after day two renewal (post-renewal). CS+; the conditioned stimulus that is paired with the aversive sound during acquisition and generalisation. CS-; the conditioned stimulus that is never
paired with an aversive sound.

Cross-modal validation

sTable 5. Within-person intraclass correlation between week one and week two for the CS differential for all studies

		Study		
**Phase**	**Stimulus**	**Validation**	**Laboratory test-retest**	**App test-retest**
**US-expectancy**				
**Acquisition**	**CS differential**	0.44 [0.10 - 0.65]	0.74 [0.52 - 0.85]	0.72 [0.45 - 0.85]
**Generalisation**	**CS differential**	0.20 [0.00 - 0.51]	0.74 [0.52 - 0.86]	0.32 [0.00 - 0.68]
**Extinction**	**CS differential**	0.56 [0.29 - 0.73]	0.49 [0.07 - 0.72]	0.20 [0.00 - 0.59]
**Renewal**	**CS differential**	0.49 [0.00 - 0.40]	0.77 [0.58 - 0.88]	0.61 [0.21 - 0.80]
**Affective ratings**				
**Baseline**	**CS differential**	0.00 [0.00 - 0.27]	0.15 [0.00 - 0.52]	0.14 [0.00 - 0.56]
**Post-extinction**	**CS differential**	0.67 [0.47 - 0.79]	0.37 [0.00 - 0.64]	0.57 [0.15 - 0.78]
**Post-renewal**	**CS differential**	0.57 [0.32 - 0.73]	0.63 [0.35 - 0.79]	0.27 [0.00 - 0.64]

Table showing the within-person 2-way absolute agreement intraclass correlations [95% confidence intervals] between week one and week two for validation, laboratory test-retest and app test-retest studies. Validation study shows intraclass correlations [95% confidence intervals] across administration mode (laboratory and app) across time (week one / week two). Laboratory and app test-retest studies show intraclass correlations [95% confidence intervals] across time (week one / week two) alone.
US-expectancy; Average self-reported US-expectancy rating per stimulus across all trials for each phase. Affective ratings; Composite affective rating comprising of self-reported feelings of anxiety, fear and unpleasantness for each stimulus at three time points i) before the experiment begins (baseline), after the extinction phase (post-extinction) and after day two renewal (post-renewal). CS differential; the difference between the CS+ conditioned stimulus that is paired with the aversive sound during acquisition and generalisation and the CS- conditioned stimulus that is never paired with an aversive sound.

US unpleasantness comparisons

We compared the self-reported unpleasantness of the scream US between app and lab administrations of the task and found that the mean rating of unpleasantness ($mean_{app}$=8.47, sd_{app} = 1.52; $mean_{laboratory}$=8.10, $sd_{laboratory}$=1.64) does not differ between the two modes of delivery (t_{68}=1.83,p=0.07). See **sTable 8** for median and range of US-unpleasantness for both modes of delivery. Individuals were excluded if they reported US-unpleasantness of 5 or less.

sTable 6. Summary of US unpleasantness by mode of delivery

	Mean	Median	St. Dev.	Min	Max
**Laboratory**	8.10	8	1.64	1	10
**App**	8.47	9	1.52	2	10

Table showing the mean, median, standard deviation and range of US-unpleasantness (1-10) rating for Laboratory and app mode of administration respectively.
St. Dev.; Standard deviation of scores

Analyses with the CS+ - CS- differential

Preliminary

sTable 7. Mean stimulus US-expectancy for CS differential in the remote validation study

**Phase**	**Mode**	**Stimulus Mean (standard error) CS +/- differential**
	**Expectancy ratings**	
**Acquisition**	**Laboratory**	5.62 (0.21)
	**App**	5.33 (0.20)
**Generalisation**	**Laboratory**	6.51 (0.21)
	**App**	6.41(0.25)
**Extinction**	**Laboratory**	1.12 (0.15)
	**App**	1.21 (0.17)
**Renewal**	**Laboratory**	2.70 (0.31)
	**App**	2.50 (0.26)
	**Affective ratings**	
**Baseline**	**Laboratory**	-0.11 (0.15)
	**App**	-0.01 (0.11)
**Post-extinction**	**Laboratory**	2.59 (0.24)
	**App**	2.10 (0.25)
**Post-renewal**	**Laboratory**	1.18 (0.24)
	**App**	1.51 (0.21)

Table showing the mean (standard error) US-expectancy rating for the CS+/ CS- differential (CS- subtracted from the CS+) stimulus averaged across all trials of each phase for laboratory and app administration for the remote validation study (n=69). Shaded portion shows the absolute difference between US-expectancy rating to the CS+ and CS- and results of t-test comparing the mean US-expectancy for the CS+ and CS- for laboratory and app administration respectively. Significant differences are indicated by a "*".

US-expectancy; Average self-reported US-expectancy rating per stimulus across all trials for each phase. Affective ratings; Composite affective rating comprising of self-reported feelings of anxiety, fear and unpleasantness for each stimulus at three time points i) before the experiment begins (baseline), after the extinction phase (post-extinction) and after day two renewal (post-renewal). CS+; the conditioned stimulus that is paired with the aversive sound during acquisition and generalisation. CS-; the conditioned stimulus that is never paired with an aversive sound.

Associations with anxiety

sFigure 6. Correlations between fear conditioning outcomes as CS differential and composite anxiety score

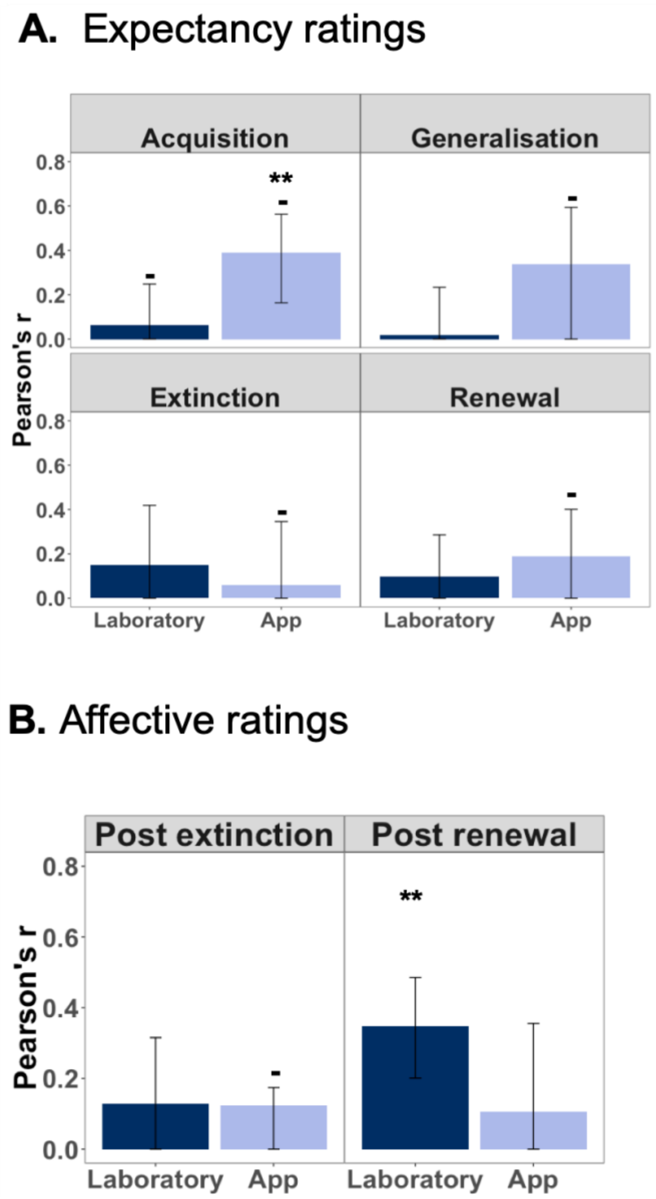

sFigure 6. Plots visualising the correlation between the mean difference between the CS+ and CS- per experimental phase and composite anxiety for the first week only in validation, app test-retest or Laboratory test-retest. Correlations presented for the app (n = 89) and Laboratory (n = 91) based testing separately. Negative correlations are indicated by the "-" symbol. Error bars represent the bootstrapped 95% confidence intervals. Significant correlations (after correcting for the effective number of independent tests) indicated by a single asterisk ("*"). **Panel A** presents bar plots showing the Pearson's correlation between average participant expectancy rating (subtracting CS- from the CS+) during acquisition, generalisation, extinction and renewal testing phases for Laboratory *(left)* and App *(right)* sessions respectively. **Panel B** presents bar plots showing the Pearson's correlations between average participant affective composite (CS- subtracted from the CS+) ratings made after the extinction phases *(Post extinction)* and after the renewal phase *(Post renewal)* for Laboratory *(left)* and App *(right)* testing sessions respectively.

Sensitivity analyses with individual anxiety measures

sFigure 7. Correlations between individual anxiety scales and fear conditioning outcomes

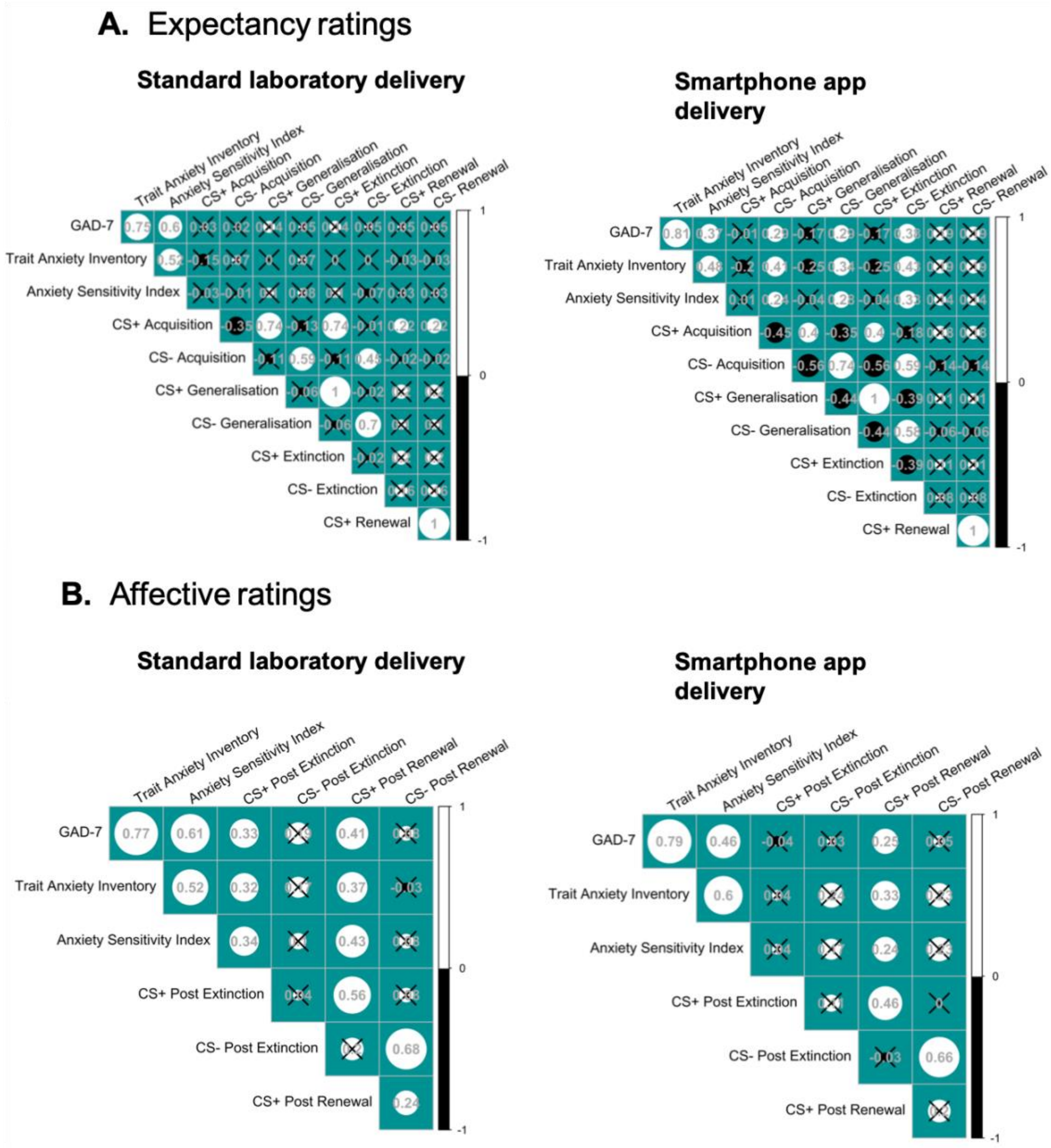

sFigure 7. Plots visualising the correlation between the three individual anxiety scales (Trait anxiety, Generalised Anxiety Disorder-7 symptoms, Anxiety Sensitivity) and fear conditioning outcome measures for first week only in validation, app test-retest or Laboratory test-retest. Correlations presented for the app (n = 89) and Laboratory (n = 91) based testing separately. **Panel A** presents heat plots showing the Pearson's correlation between participant anxiety measures and fear expectancy during acquisition, generalisation, extinction and renewal testing phases for Laboratory (*left)* and App *(right)* sessions respectively. **Panel B** presents bar plots showing the Pearson's correlations between participant anxiety measures and affective composite ratings made after the extinction phases (Post extinction) and after the renewal phase (Post renewal) for Laboratory *(left)* and App *(right)* testing sessions respectively.

Colour of the circle indicates direction of the association (white = positive, black = negative), size of the circle indicates the strength of the relationship (larger circles indicate higher Pearson's r value). Non-significant correlations (P > 0.05) are crossed out using a large black 'x' symbol.

References

Peterson, R. A., & Heilbronner, R. L. (1987). The anxiety sensitivity index: *Journal of Anxiety Disorders*, *1*(2), 117–121. doi:10.1016/0887-6185(87)90002-8

Spielberger, C. D. (1983). *STAI State-trait Anxiety Inventory for Adults Form Y: Review Set; Manual, Test, Scoring Key.* Mind Garden.

Spitzer, R. L., Kroenke, K., Williams, J. B. W., & Löwe, B. (2006). A brief measure for assessing generalized anxiety disorder: the GAD-7. *Archives of Internal Medicine*, *166*(10), 1092–1097. doi:10.1001/archinte.166.10.1092
